# Tryptophan catabolites and depression in the general population: results from the Gutenberg Health Study

**DOI:** 10.1186/s12888-023-04520-6

**Published:** 2023-01-11

**Authors:** Matthias Michal, Andreas Schulz, Philipp S. Wild, Thomas Koeck, Thomas Münzel, Alexander K. Schuster, Konstantin Strauch, Karl Lackner, Sigurd D. Süssmuth, Heiko G. Niessen, Andreas Borta, Kelly A. Allers, Daniela Zahn, Manfred E. Beutel

**Affiliations:** 1 Department of Psychosomatic Medicine and Psychotherapy, University Medical Center of the Johannes Gutenberg University Mainz, Langenbeckstr. 1, 55131 Mainz, Germany; 2 German Center for Cardiovascular Research (DZHK), Partner Site Rhine-Main, University Medical Center of the Johannes Gutenberg University, 55131, Langenbeckstr. 1, Mainz, Germany; 3 Preventive Cardiology and Preventive Medicine, Center for Cardiology, University Medical Center of the Johannes Gutenberg University Mainz, Mainz, Germany; 4 Center for Cardiology – Cardiology I, University Medical Center of the Johannes Gutenberg University, Mainz, Germany; 5 Department of Ophthalmology, University Medical Center of the Johannes Gutenberg-University Mainz, Mainz, Germany; 6 Institute of Medical Biostatistics, Epidemiology and Informatics (IMBEI), University Medical Center, Johannes Gutenberg University, Mainz, Germany; 7 Institute of Clinical Chemistry and Laboratory Medicine, University Medical Center of the Johannes Gutenberg University Mainz, Mainz, Germany; 8 Clinical Development, uniQure NV, Allschwil, Switzerland; 9 Department of Neurology, Univeristy Hospital of Ulm University, Ulm, Germany; 10 Department of Translational Medicine & Clinical Pharmacology, Boehringer Ingelheim Pharma GmbH & Co. KG, Birkendorfer Strasse 65, 88397 Biberach an Der Riss, Germany; 11 CNS Diseases Research, Boehringer Ingelheim Pharma GmbH & Co. KG, Birkendorfer Strasse 65, 88397 Biberach an Der Riss, Germany

**Keywords:** Tryptophan, Depression, Kynurenine pathway

## Abstract

Previous studies reported significantly altered tryptophan catabolite concentrations in major depression. Thus, tryptophan catabolites were considered as potential biomarkers of depression and their modulators as potential targets for psychopharmacotherapy. However, the results were based mainly on studies with small sample sizes limiting their generalizability. Against this background, we investigated the relationship of peripheral tryptophan catabolites with depression in a population-based sample with *n* = 3,389 participants (with fasting status ≥ 8 h and C-reactive protein < 10 mg/L). *N* = 248 had clinically significant depression according to a PHQ-9 score of ≥ 10, *n* = 1,101 subjects had mild depressive symptoms with PHQ-9 scores between 5 and 9, and n = 2,040 had no depression. After multivariable adjustment, clinically significant depression was associated with lower kynurenine and kynurenic acid. Spearman correlation coefficients of the tryptophan catabolites with the severity of depression were very small (rho ≤ 0.080, *p* ≤ 0.015). None of the tryptophan catabolites could diagnostically separate depressed from not depressed persons. Concerning linear associations, kynurenine and kynurenic acid were associated only with the severity and the cognitive dimension of depression but not its somatic dimension. Tryptophan catabolites were not associated with persistence or recurrence of depression at the 5 year follow-up. The results replicated the association between kynurenine and kynurenic acid with depression. However, the associations were small raising doubts about their clinical utility. Findings underline the complexity of the relationships between depression and tryptophan catabolites. The search for subgroups of depression with a potentially higher impact of depression might be warranted.

## Background

The metabolism of tryptophan, the precursor of the neurotransmitters serotonin and melatonin, has been considered as one of the essential biological pathways of depression [1, 2]. The catabolism of tryptophan has two major enzymatic pathways. For the etiology of depression, the pathway involving the Indoleamine-2,3-dioxygenase (IDO1) has been assumed to play a major role. Proinflammatory cytokines can activate IDO1. Tryptophan is catabolized to kynurenine, the neuroprotective kynurenic acid, and the neurotoxic quinolinic acid [3].

However, tryptophan metabolism is also closely related to inflammation and inflammatory diseases such as infections, coronary heart disease, autoimmune syndromes, cancer, and neurodegenerative disorders [4, 5]. These inflammatory diseases are often comorbid with major depression and thus might distort findings on the relationship of tryptophan metabolism with mental disorders [6, 7]. Further, the current knowledge on the relationship between tryptophan metabolism and depression is mainly based on small clinical samples, which can affect the generalizability of the results.

Previous studies suggested that the kynurenine pathway might play a crucial role in the etiology of depression. Blood tryptophan catabolites were seen as candidates for detecting depression, differentiating subtypes, predicting treatment response or pathways for novel treatments. Plasma levels of kynurenic acid, quinolinic acid, and associated ratios separated patients with a diagnosis of major depression and healthy controls and yielded a high diagnostic accuracy of above 70% for kynurenic acid [8]. The area under the curve for tryptophan levels was 0.74 for the detection of major depression [9]. In another study the increase of kynurenic acid after the first infusion of ketamine was strongly associated with antidepressant response [10]. Antidepressant-like effects of kynurenic acid were also reported by an animal study based on reversing immobility and improving climbing and swimming times in a forced swimming test [11]. Kynurenic acid has also been assumed to play a role in cognitive functioning. In late-life depression, elderly depressed persons with memory deficits had significantly lower blood concentration of kynurenic acid compared to those without memory deficits [12]. Conversely, in a recent study on post-stroke depression, serum kynurenic acid levels correlated positively with depression [13].

Meta-analyses on the kynurenine pathway reported decreases in kynurenic acid and kynurenine levels and increases in quinolinic acid levels in patients with depression [14], respectively found lower concentration of kynurenine in unipolar major depression versus healthy controls [3]. A recent meta-analysis postulated that tryptophan and kynurenic acid were consistently downregulated in patients with major depression, regardless of exposure to antidepressants [15]. Moreover, the authors suggested that tryptophan and kynurenic acid demonstrated the highest potential for distinguishing states of depression and treatment response [15]. Some researchers have therefore postulated that the control of kynurenic acid production might be a target for the drug treatment of depression and other brain disorders characterized by neuroinflammation [15–17]. However, among current studies, there was much heterogeneity. Most studies had only tiny sample sizes or considered only a small range of the kynurenine pathway and potential covariates.

Yet, depression is not a one-dimensional construct [16]. The Patient Health Questionnaire PHQ-9, measures a somatic (sleeping problems, fatigability, appetitive problems, and psychomotor retardation) and a cognitive-affective dimension (lack of interest, depressed mood, negative feelings about self, concentration problems, and suicidal ideation). These have been differentially related to medical outcomes [17]. Therefore, the purpose of this trial is also to determine the associations of tryptophane catabolites to the cognitive-affective and somatic dimensions of depression.

Against this background, our study of a large population-based cohort has several aims. First, to examine whether circulating concentrations of tryptophan catabolites are different between subjects with and without current depression. Second, we wanted to replicate whether tryptophan catabolites have sufficient diagnostic potential in a population-based sample. Third, we investigated linear associations between tryptophan catabolites and dimensions, respectively, severity of depression.

## Methods

### Sample

This study analyzed data from the first *n* = 5,000 participants in the Gutenberg Health Study (GHS). The actual sample being analyzed was reduced to *n* = 3,389, because of the exclusion of persons with a fasting time < 8 h (1298), current infections (determined by CRP ≥ 10 mg/L; *N* = 154), missing depression score and missing data in any other variable (*N* = 114). Persons with current infections and non-fasting status were excluded, because these conditions have an impact on peripheral tryptophan catabolites.

The GHS is a population-based, prospective, observational single-center study in western Mid-Germany with an age range of 35 to 74 years. Exclusion criteria were insufficient German language skills and physical and mental disability to participate. The sample had been stratified for sex, residence, and decades of age. The study protocol and study documents were approved by the local ethics committee of the Medical Chamber of Rhineland-Palatinate, Germany (reference no. 837.020.07; original vote: 22.3.2007, latest update: 20.10.2015) and by the local and federal data safety commissioners.

### Assessment

Participants underwent an examination of a five-hour duration in the study center, including questionnaires, computer-assisted personal interviews, laboratory, and medical examinations. A five-year follow-up assessment was conducted in the study center with a similar, comprehensive assessment.

The tryptophan catabolites were analyzed by a qualified LC–MS/MS assay at Nuvisan, Neu-Ulm, Germany. The analytes were extracted from plasma after addition of the internal standards [D5] kynurenic acid, [D4] kynurenine, [D5] tryptophan and [D3] quinolinic acid and protein precipitation followed by chromatographic separation on an ultra-performance liquid chromatography column and mass spectrometric detection using positive multiple-reaction monitoring on two mass analyzers. In detail, for mass spectrometry AB Sciex API 5000™ System, and for liquid chromatography Waters Acquity LC System was used, using a Force C18 column (3 μm, 50 × 3.0 mm; Restek). Mobile phase B consisted of 2% formic acid in water, and mobile phase A consisted of acetonitrile/methanol/2-propanol/formic acid (46.5/46.5/5/2, v/v/v/v). Plasma samples were prepared by the addition of internal standards working solution followed by ice-cold methanol for protein precipitation. Subsequently, samples were centrifuged and the supernatant was evaporated to dryness under a stream of nitrogen and reconstituted in 150 µL take up solution (water/acetonitrile/formic acid/ascorbic acid (97.4 mL/ 2 mL/ 0.5 mL/ 100 mg)). In-study coefficients of variation for precision were determined for tryptophan catabolites as follows: Tryptophan (3.9% – 6.1%), kynurenine (3.7% – 5.1%), kynurenic acid (5.2%—8.4%) and quinolinic acid (4.3%—5.9%). Lower limits of quantification in plasma were: 2000 nmol/L for tryptophan, 100 nmol/L for kynurenine, 5 nmol/L for kynurenic acid, and 50 nmol/L for quinolinic acid.

Depression was assessed by the 9 item depression module of the Patient Health Questionnaire (PHQ-9) at baseline and at the five-year follow-up. A cut-off score ≥ 10 determined depression (Cronbach’s α, 0.80, sensitivity 81%, and specificity 82% for detecting any depressive disorders [18]. The PHQ-9 comprises somatic and cognitive dimensions of depression. Items related to problems with sleep, lack of energy, appetite, and psychomotor agitation/retardation represent somatic depressive symptoms. The remaining five items, measuring lack of interest, depressed mood, negative feelings about self, problems with concentration, and suicidal ideation represent the cognitive dimensions of depression [19]. Anxiety was measured by the 2 item version of the General Anxiety Disorder questionnaire (GAD-2). The total GAD-2 score has a range from 0 to 6. Using a cut-off score of 3 or more, the GAD-2 identifies any anxiety disorder (e.g., generalized anxiety disorder, social phobia, or panic disorder) with a sensitivity of 65% and specificity of 88% [20].

Socioeconomic status (SES) was defined according to Lampert and Kroll as ranging from 3 (lowest) to 21 (highest). The multidimensional index combines information about educational qualifications, household characteristics of occupation, and income with equal weights [21].

The history of any suicide attempt was assessed at the 5-year follow-up by self-report. During the computer-assisted personal interview, participants were asked whether they had ever received a definite diagnosis of any depressive or anxiety disorder by a physician or psychotherapist (medical history of depression/ anxiety disorder). Smoking was dichotomized into smokers and non-smokers (never smoker and ex-smoker), obesity was defined as a body mass index (BMI) ≥ 30. At risk consumption of alcohol was defined as daily consumption of ≥ 24 mg for men and ≥ 12 mg for women. The level of physical activity expressed as metabolic equivalents (MTS) was assessed with the Short Questionnaire to Assess Health-enhancing physical activity (SQUASH) [22].

Blood samples were taken in fasting conditions. Serum lipid levels (total cholesterol, triglycerides, and high-density lipoprotein cholesterol), plasma concentration of C-reactive protein were measured immediately after blood withdrawal by routine methods; low-density lipoprotein cholesterol was calculated by the Friedewald formula. All other measurements were determined in plasma or serum stored immediately after blood withdrawal and centrifugation at − 80 °C until analysis. The measurements were done in a blinded fashion in a single batch. Insulin resistance (HOMA-IR, homeostasis model assessment-insulin resistance) was calculated using the following formula: fasting insulin (µU/ml) × fasting glucose (mmol/l) divided by 22.5.

Medication was registered on-site by scanning the bar codes of the original packages of drugs taken by participants. Active ingredients were recorded using the Anatomical Therapeutic Chemical (ATC) Classification System. Three classes of antidepressants were noted: nonselective monoamine reuptake inhibitors (ATC N06AA), selective serotonin reuptake inhibitors (ATC N06AB), and other antidepressants (ATC N06AX).

### Statistical analyses

Variables were reported as numbers/percentages, means (± standard deviation) or medians (and interquartile range (25th/75th) as appropriate. For the analyses, the sample was divided into three groups based on the baseline depression score: A sample of participants with PHQ-9 ≥ 10, indicating current moderate to severe depression. The control group comprised persons without clinically significant depressive symptoms as indicated by PHQ-9 < 5 and lack of a prior history of depression or anxiety disorder or intake of any psychiatric medication. As alterations of the tryptophan catabolites could also cause mild depressive symptoms [5], we also included an intermediate group of persons with PHQ-9 scores between 5 to 9 or a previous history of depression/anxiety or intake of any psychiatric drugs. The group of persons with PHQ-9 ≥ 10 and the control group were used for categorical comparisons, and the complete sample was used for analyzing linear associations of tryptophan catabolites with the severity of depression.

First, we compared the group of not depressed participants with the group of depressed participants. The tryptophan catabolites were compared between the groups of persons with and without depression by t-tests in the unadjusted analysis. Second, we calculated logistic regression analyses with the dependent variable depression (PHQ-9 ≥ 10) versus no depression (PHQ-9 < 5) and one of the tryptophan catabolites as the predictor and three models of adjustment (model 1: sex, age; model 2: sex, age, SES; model 3: sex, age, SES, smoking, obesity, alcohol abuse, physical activity score, heart rate, systolic blood pressure [mmHg], c-reactive protein (CRP), insulin resistance (HOMA-IR). Third, we calculated ROC curve to evaluate the efficacy of the tryptophan catabolites for diagnosing depression as determined by a PHQ-9 ≥ 10. In the next step, we analyzed the linear associations of tryptophan catabolites with the severity of depression as determined by the PHQ-9 sum score (range 0–27). Forth, we calculated a Spearman rank correlation of depression with the tryptophan catabolites. Fifth, we calculated a linear regression analysis with the dependent variable severity of depression and its subcomponents with the predictor kynurenic acid and the covariates sex, postmenopausal status, age, blood pressure, coronary artery disease, heart rate, body mass index, smoking, high-density lipoprotein, low-density protein, triglycerides, C-reactive protein, insulin resistance (HOMA-IR), physical activity, and alcohol abuse.

## Results

The characteristics of the sample are shown in Table 1. As compared to the group without current and previous depression, the group of depressed persons (PHQ-9 ≥ 10) had a lower mean age and a higher proportion of females, particularly postmenopausal women. Forty-four percent of depressed persons had a medical history of depression, and more than 12% a history of suicide attempts. Their SES was lower, and they were less often living in a partnership. Concerning other medical diseases, they had higher rates of arthritis and chronic obstructive pulmonary disease. They were more often obese and had higher smoking rates. Concerning biological markers, they had a higher CRP level and lower systolic blood pressure. At the five-year follow-up, 52.9% of the depressed group had PHQ-9 scores ≥ 10.

**Table 1:** Characteristics of the sample by depression severity

	No Depression (PHQ-9 < 5) *n* = 2040	Depression (PHQ-9 ≥ 10) *n* = 248	Mild depression (PHQ-9 = 5–9) *n* = 1101
Women, % (n)	42.6% (870)	60.1% (149)	57.2% (630)
postmenopausal Women, % (n)	28.7% (586)	41.1% (102)	39.7% (436)
Age in years, mean ± sd	55.2 ± 10.9	53.2 ± 10.5	55.1 ± 10.9
Socioeconomic status (SES) (3 = lowest SES, 21 highest SES), mean ± sd	13.20 ± 4.41	11.68 ± 4.12	12.04 ± 4.29
Partnership, % (n)	85.8% (1751)	72.6% (180)	79.5% (873)
PHQ-9, median (IQR)	2.00 (1.00/3.00)	11.00 (10.00/13.00)	6.00 (5.00/7.00)
Somatic symptom dimension of depression	1.00 (0/2.00)	6.00 (5.00/7.00)	3.00(2.00/4.00)
Cognitive symptom dimension of depression	0 (0/1.00)	6.00(4.00/7.00)	2.00(1.00/3.00)
GAD-2, median (IQR)	0 (0/1.00)	2.00 (2.00/4.00)	1.00 (0/2.00)
History of depression, % (n)	/	44.0% (109)	23.6% (259)
History of suicide attempt, % (n)	0.8% (13)	12.4% (23)	3.6% (32)
History of any anxiety disorder, % (n)	/	22.2% (55)	14.3% (157)
Depression (PHQ-9 ≥ 10) at the 5 –year follow-up	2.4% (42)	52.9% (100)	11.8% (108)
**Psychiatric medication**			
Antidepressants, % (n)	/	18.1% (45)	10.7% (117)
Antipsychotics, % (n)	/	2.4% (6)	1.6% (17)
Anxiolytics, % (n)	/	4.0% (10)	2.8% (31)
Hypnotics and sedatives, % (n)	/	4.0% (10)	3.6% (39)
**Physical Disease**			
Diabetes mellitus, % (n)	8.7% (176)	12.2% (30)	8.6% (95)
Arterial Hypertension, % (n)	51.9% (1058)	46.4% (115)	47.0% (518)
Dyslipidemia, % (n)	34.6% (706)	40.7% (101)	33.9% (372)
Peripheral artery disease, % (n)	3.7% (76)	5.6% (14)	5.1% (55)
Coronary artery disease, % (n)	3.5% (70)	5.7% (14)	5.4% (58)
Chronic heart failure, % (n)	1.4% (28)	1.6% (4)	1.2% (13)
Chronic obstructive pulmonary disease, % (n)	3.7% (75)	5.6% (14)	5.8% (64)
Chronic liver disease, % (n)	0.8% (16)	1.2% (3)	0.6% (7)
Chronic kidney disease,, % (n)	0.5% (11)	0.8% (2)	1.2% (13)
Cancer, % (n)	8.4% (171)	9.7% (24)	10.4% (114)
Arthritis, % (n)	4.1% (83)	8.5% (21)	5.7% (63)
**Lifestyle factors**			
Smoking, % (n)	17.5% (357)	27.4% (68)	20.3% (223)
Physical activity score [in 1000]	7.12 (4.81/9.39)	7.13 (5.01/9.25)	7.04 (4.97/9.50)
Alcohol abuse [24/12 g/d], % (n)	23.9% (488)	22.6% (56)	24.8% (273)
Obesity, % (n)	22.1% (450)	25.4% (63)	25.0% (275)
**Biomarkers**			
C-Reactive Protein (CRP, mg/L)	1.60 (0.50/2.90)	1.80 (1.10/3.50)	1.60 (1.00/3.20)
Insulin resistance (HOMA-IR)	1.75 (1.25/2.58)	1.76 (1.23/2.58)	1.76 (1.25/2.53)
Cholesterol [mmol/L]	224.2 ± 41.3	224.2 ± 43.9	224.4 ± 41.7
Systolic blood pressure [mmHg]	133.4 ± 18.0	129.6 ± 16.0	130.2 ± 17.3
Heart rate [beats per minute]	68.1 ± 10.9	68.1 ± 10.6	68.7 ± 10.7

### Concentration of tryptophan catabolites

Table 2 displays the comparison of the levels of tryptophan catabolites of persons with versus without depression. In the crude comparison, there was a significant difference for kynurenine and kynurenine acid. In the fully adjusted logistic regression analyses (model 3), kynurenine and kynurenine acid were still lower in the depressed versus not-depressed group.

**Table 2:** Comparison of the concentration of tryptophan catabolites and prediction of depression by tryptophan catabolites

	Not depressed (PHQ-9 < 5) *n* = 2040	Depressed (PHQ-9 ≥ 10) *n* = 248	Estimate (diff. 95% CI), p	Test model 1 OR (95% CI), *p*	Test model 2 OR (95% CI), *p*	Test model 3 OR (95% CI), *p*
Tryptophan [nmol/l]	50,188 ± 9121	49,038 ± 10,214	1150 [-186; 2487], *p* = 0.091	1 (1–1) *p* = 0.97	1 (1–1) *p* = 0.82	1 (1–1) *p* = 0.79
Kynurenine [nmol/l]	2570 (2180/3040)	2445 (2060/2920)	-0.23 [-0.096; -0.36], *p* = 0.00077	0.905 (0.798–1.025) *p* = .12	0.905 (0.798–1.026) *p* = .12	0.861 (0.753–0.984) *p* = 0.028
Kynurenic acid [nmol/l]	33.90 (27.50/42.36)	32.00 (24.44/38.70)	-0.27 [-0.12; -0.42], *p* = 0,00,045	0.814 (0.715 -0.925) *P* = 0.0016	0.827 (0.727–0.939) *p* = 0.0037	0.834 (0.731–0.951) *p* = 0.0071
Quinolinic acid [nmol/l]	309.5 (253.0/384.0)	296.5 (240.0/378.3)	-0.097 [-0.046; 0.24], *p* = 0.18	0.952 (0.836 -1.081) *p* = 0.46	0.946 (0.830–1.075) *p* = 0.40	0.917 (0.796–1.053) *p* = 0.23

Student’s t-test for the comparison of tryptophan catabolite concentration between not depressed and depressed persons. Tryptophan catabolite (trycat) concentrations are shown as means (± standard deviation) or medians (and interquartile range (25th/75th)

The odds ratios reflect change per standard deviation for log-transformed kynurenine, kynurenine acid and quinolinic acid. Tryptophan was normally distributed and thus not log-transformed

Test model 1 = logistic regression analysis with the dependent variable depressed versus not depressed and the independent variables trycat per standard deviation and sex and age. For model 1, *n* = 248 cases of depression could be used

Test model 2 = logistic regression analysis with the dependent variable depressed versus not depressed and the independent variables trycat per standard deviation and sex, age, SES. For model 2, *n* = 246 cases of depression could be used

Test model 3: logistic regression analysis with the dependent variable depressed versus not depressed and the independent variables trycat per standard deviation and sex, age, SES, smoking, obesity, excessive alcohol consumption, physical activity score, heart rate, systolic blood pressure [mmHg], CRP, HOMA-IR, LDL/HDL. For model 3, *n* = 187 cases of depression could be used

We also calculated a logistic regression analysis with the outcome clinically significant depression (PHQ-9 ≥ 10) at the 5-year follow-up and the predictor log-transformed kynurenic acid and the covariates PHQ-9 score at baseline, age, and sex. Kynurenic acid, however, was not associated with later depression (estimate—0.0216, 95% CI -0.136/0.0923, *p* = 0.71).

### Receiver operating characteristics of tryptophan catabolites for diagnosing depression

The ROC for the detection of depression according to PHQ-9 ≥ 10 by tryptophan catabolites was not significant. The Area under the curve (AUC) for kynurenine, kynurenic acid, quinolinic acid, and tryptophan were 0.472 (0.376/0.567), 0.410 (0.315/0.505), 0.462 (0.362/0.563), and 0.413 (0.319/0.506).

### Correlates of kynurenic acid

First, we calculated Spearman rank correlations of the tryptophan catabolites with the PHQ-9 sum score. The coefficients were very low and ranged between rho =—0.042 (for quinolinic acid, *p* = 0.015) and rho—0.080 (for kynurenic acid, *p* < 0.0001). Second, we calculated linear regression analyses with the dependent variables severity of depression and its subcomponents, cognitive and somatic symptoms of depression, and the predictors kynurenine and kynurenic acid and 20 covariates. Kynurenine and Kynurenic acid were not independently associated with the severity of depression or the somatic symptom dimension of depression. However, kynurenine and kynurenic acid were independently associated with the severity of cognitive symptoms of depression (Table 3).

**Table 3:** Linear regression with the dependent variables severity of depression according to the sum score of PHQ-9 and its subcomponents somatic and cognitive symptoms of depression

	**Severity of depression (PHQ-9)** R^2^ = 0.054, *n* = 3764				**somatic symptoms of depression** R^2^ = 0.059, *n* = 3762				**cognitive symptoms of depression** R^2^ = 0.038, *n* = 3761			
	Estimate	95% CI		***p***	Estimate	95% CI		***p***	Estimate	95% CI		***p***
Kynurenine	-0.186	-0.332	-0.0398	**0.013**	-0.0455	-0.127	-0.036	0.28	-0.141	-0.222	-0.591	**0.00072**
Kynurenic acid (per log SD)	-0.280	-0.475	-0.08416	**.0051**	-0.0890	-0.198	0.025	0.11	-0.139	-0.209	-0.0683	**0.00011**

Adjusted for sex, menopause (postmenopausal), time of blood sampling [h], age (in years), systolic blood pressure [mmHg], diastolic blood pressure [mmHg], heart rate [beats per minute], BMI [kg/m^2^], smoking (yes), coronary artery disease (yes), HDL [mg/dl], LDL [mg/dl], triglycerides [mg/dl], CRP [mg/L], insulin resistance (HOMA-IR), physical activity score [in 1000 METS], at-risk consumption of alcohol (> 24/12 g/d) (yes)

## Discussion

In this large population-based sample, we found differences in tryptophan catabolites between depressed persons and non-depressed controls in accordance with the latest meta-analysis [2]. Depressed versus non-depressed persons had lower peripheral concentrations of kynurenine and kynurenic acid. This finding was robust for multiple adjustments. In contrast to the meta-analysis of Marx et al., we found no significant difference for tryptophan [2]. Further, our results contrast with a recent analysis from the community-based Netherlands Study of Depression and Anxiety [23], where no differences in plasma tryptophan catabolites could be found comparing *n* = 1100 persons with major depression with *n* = 642 healthy controls.

The prominent role of kynurenine and kynurenic acid, which we found in our study, is in line with previous studies assuming that control of kynurenic acid might be a major target for antidepressant drug development [11, 24–27]. Furthermore, regarding the association with depression, we demonstrated for the first time a specific association of kynurenine and kynurenic acid with different symptom clusters of depression. Both catabolites were not associated with somatic symptoms of depression (e.g., problems with sleep, lack of energy). However, there was an independent inverse association of the neuroprotective kynurenine and kynurenic acid with the cognitive-affective symptom dimension of depression (i.e., depressed mood, loss of interest, difficulties concentrating, suicidal ideation). The finding of such a differential association is consistent with previous studies, demonstrating a specific role of kynurenine and kynurenic acid for anhedonia and suicidal ideation [10, 25, 28]. Though our finding contrasted with the recent analysis from the Netherlands Study of Depression and Anxiety, in which kynurenic acid correlated inversely with melancholic features (mood worse in the morning, early morning awakening, distinct quality of mood, excessive guilt, decreased appetite, decreased weight, psychomotor agitation, and psychomotor retardation) [23]. Although different scales might hamper comparability, melancholic features correspond more to the somatic symptom dimension than the cognitive one. All things considered, the effect sizes we found were very small, so replicating the results would certainly make sense.

In contrast to previous reports [8, 9, 15] from highly selected samples, we could not replicate the usefulness of tryptophan catabolites as screening markers for the detection of depression on a population-level. The area under the curve was very close to the diagonal non-discrimination line. Therefore, these biomarkers appear unsuitable for diagnosing depression or monitoring depression status in a real-world sample. This negative finding is not surprising because tryptophan catabolites play a role in many different diseases and hence may be too unspecific for screening depression [29, 30]. Further, another plausible explanation for this negative finding is that we used a population-based sample instead of a clinical sample with presumably lower contrast between the groups.

A limitation of our study is that depression status was questionnaire-based and not determined by a clinical interview. However, the PHQ-9 is a valid instrument for measuring the severity of depression and carrying important prognostic information [31–33]. Another limitation of our study is that we relied on peripheral tryptophan catabolites only, as there might be relevant differences between central and peripheral tryptophan catabolites. Exclusion of 1298 participants due to fasting time < 8 h may have biased findings. A recent study showed that in bipolar patients with ongoing depressive symptoms, kynurenic acid concentrations were decreased in the plasma but unchanged in the cerebrospinal fluid [34]. Other studies reported that plasma tryptophan catabolites are an excellent proxy of the kynurenine pathway in the cerebrospinal fluid of depressed patients [25]. A systematic review found moderate to strong concordance between peripheral concentrations of kynurenine but less evidence for other tryptophan metabolites [35, 36].

In summary, we confirmed the association of the tryptophan catabolites kynurenine and kynurenic acid with depression in a large population-based sample. After multivariable adjustment, clinically significant depression was only predicted by kynurenine and kynurenic acid but not the other tryptophan catabolites. A novel finding was that kynurenine and kynurenic acid predicted the overall severity and the cognitive-affective dimension of depression but not its somatic dimension. However, the associations found were small, raising doubts about their clinical utility. Findings underline the complexity of the relationships between depression and tryptophan catabolites. Given that a large study including community controls found no association [25] our findings might stimulate the search for subgroups of more severe depression where tryptophan catabolites have stronger effects.

## Data Availability

Written informed consent from GHS study participants does not allow public access to the data. Access to the data in the local database is possible at any time upon request according to the ethics vote. This concept was developed with the local data protection officer and the ethics committee (local ethics committee of the Rhineland-Palatinate Medical Association, Germany). Interested scientists can make their requests to the Gutenberg Health Study Steering Committee (e-mail: info@ghs-mainz.de).

## Abbreviations

IDO1: Indoleamine-2,3-dioxygenase
GHS: Gutenberg Health Study
UPLC: Ultra Performance Liquid Chromatography
PHQ-9: Patient Health Questionnaire, depression module
GAD-2: General Anxiety Disorder questionnaire
SES: Socioeconomic status
SQUASH: Questionnaire to Assess Health-enhancing physical activity
HOMA-IR: Homeostasis model assessment-insulin resistance
MTS: metabolic equivalents
CRP: C-reactive Protein
